# Bone marrow mesenchymal stem cell exosomes suppress JAK/STAT signaling pathway in acute myeloid leukemia in vitro

**DOI:** 10.1007/s44313-024-00051-5

**Published:** 2024-12-20

**Authors:** Sahar Jalilivand, Maryam Nabigol, Mehdi Bakhtiyaridovvombaygi, Ahmad Gharehbaghian

**Affiliations:** 1 Department of Laboratory Hematology and Blood Bank, School of Allied Medical Sciences, Shahid Beheshti University of Medical Sciences, Tehran, Iran; 2 Student Research Committee, Department of Hematology and Blood Banking, School of Allied Medical Sciences, Shahid Beheshti University of Medical Sciences, Tehran, Iran; 3 Hematopoietic Stem Cell Research Center, Shahid Beheshti University of Medical Sciences, Tehran, Iran

**Keywords:** Bone marrow mesenchymal stem cell, Exosome, Acute myeloid leukemia, JAK2, STAT3, STAT5

## Abstract

**Introduction:**

Despite advances in the treatment of acute myeloid leukemia (AML), refractory forms of this malignancy and relapse remain common. Therefore, development of novel, synergistic targeted therapies are needed urgently. Recently, mesenchymal stem cells (MSCs) have been shown to be effective in treating various diseases, with most of their therapeutic outcomes attributed to their exosomes. In the current study, we investigated the effects of bone marrow mesenchymal stem cell (BM-MSC) exosomes on the expression of the Janus kinase/signal transducers and activators of transcription (JAK/STAT) signaling genes involved in AML pathogenesis.

**Material and Methods:**

Exosomes were isolated from BM-MSCs and confirmed using transmission electron microscopy, dynamic light scattering, and flow cytometry. Subsequently, the exosome concentration was estimated using the bicinchoninic acid assay, and HL-60 cells were cocultured with 100 µg/mL of BM-MSC exosomes. Finally, the JAK2, STAT3, and STAT5 expression levels were analyzed using qRT-PCR.

**Results:**

The exosome characterization results confirmed that most isolated nanoparticles exhibited a round morphology, expressed CD9, CD63, and CD81, which are specific protein markers for exosome identification, and ranged between 80 and 100 nm in diameter. Furthermore, qRT-PCR analysis revealed a significant downregulation of JAK2, STAT3, and STAT5 in HL-60 cells treated with 100 μg/mL of BM-MSC exosomes.

**Conclusion:**

Since JAK/STAT signaling contributes to AML survival, our findings suggest that the downregulation of JAK/STAT genes by BM-MSC exosomes in leukemic cells may aid in designing a potent therapeutic strategy for AML treatment.

## Introduction

Acute myeloid leukemia (AML) is a widely prevalent heterogeneous disease characterized by extreme clonal proliferation of hematopoietic precursors and their differentiation arrest [1, 2]. It ranks among the top 15 cancers with the highest prevalence and accounts for 1% of all new cancer cases annually and approximately 80% of all leukemia cases in adults. AML incidence increases with age, with a median age of 67 years at diagnosis [3, 4].

The primary goal of AML treatment is to achieve complete remission (CR) with induction chemotherapy. However, patients with AML experience a significant failure rate in achieving CR and are prone to relapse in a way that approximately 50% to 70% of patients with AML who achieve CR through initial therapy face relapse within three years. Consequently, consolidation chemotherapy or allogeneic stem cell transplantation is often used to prevent relapse [4, 5].

From in vitro experiments to in vivo animal models and clinical trials, mesenchymal stem cells (MSCs) have shown significant potential in treating various disorders, including leukemia [6, 7]. MSCs constitute a group of non-hematopoietic stem cells that were initially discovered in the bone marrow, which is now acknowledged as their primary source [8]. These cells can modulate numerous cellular pathways through direct cell-to-cell interaction or by secreting a variety of factors such as exosomes, growth factors (e.g., vascular endothelial growth factor (VEGF)), interleukins (including IL-6 and −8), and cytokines (such as transforming growth factor-β (TGF-β)) [9, 10].

Recently, there has been an increasing focus on exosomes as vital intercellular communication vehicles, first discovered in 1983 [11]. Exosomes, recognized as essential mediators of MSC function, are nanosized (30–200 nm), single-membraned, secreted extracellular vesicles capable of delivering cargo (e.g., nucleic acids, proteins, lipids, and other biomolecules) from donor to recipient cells, influencing recipient cell's viability, proliferation, programmed cell death, and drug sensitivity [12, 13].

As a pivotal component of numerous critical cellular processes, the JAK/STAT pathway constitutes a rapid membrane-to-nucleus signaling module that is activated by various ligands, including cytokines, growth factors, and hormones, and induces the expression of many essential cancer mediators [14, 15].

JAK/STAT is a central cancer pathway, and its hyperactivation plays a critical role in AML pathogenesis [16, 17]. Structurally, this pathway involves JAKs (JAK1, JAK2, JAK3, and TYK2), STATs (STAT1, STAT2, STAT3, STAT4, STAT5a, STAT5b, and STAT6), and ligand-receptor complexes [18]. In this cascade, JAK transphosphorylation and subsequent activation, triggered by ligand binding to the receptor, induce tyrosine phosphorylation of the receptor. This event creates a docking site at which JAK phosphorylates STAT. Subsequently, phosphorylated STAT dissociates from the receptor to form homo/heterodimers. These dimers are translocated to the nucleus, bind to DNA, and regulate the transcription of target genes [19–21].

Growing evidence indicates that dysregulation of the JAK/STAT pathway is associated with various types of cancer, and its constant activation by mutations or overexpression in myeloid blasts highlights the involvement of this signaling pathway in the malignant transformation of AML blasts [14, 22].

In addition to tumorigenesis and progression, studies have revealed that JAK/STAT mutations mediate drug resistance in AML [23]. Therefore, inhibition of the JAK/STAT pathway to impede leukemic cell proliferation may play a significant role in targeted therapy for AML [22].

Our study aimed to investigate the effect of bone marrow mesenchymal stem cell (BM-MSC) exosomes on AML and to explore whether these exosomes could regulate the expression of JAK2, STAT3, and STAT5 in HL-60 cells, which have not been widely reported elsewhere, as these proteins are known to play critical roles in leukemogenesis by regulating cell survival, proliferation, apoptosis, and drug response [4, 16]. We hypothesized that BM-MSC exosomes suppress AML by reducing the expression of JAK2, STAT3, and STAT5 in HL-60 cells.

## Methods and materials

### Cell Lines, cultivation methods, and identification

Human leukemia cell line (HL-60 cells) was obtained from the Pasteur Institute (Tehran, Iran) at passage 15. The cells were maintained in the Roswell Park Memorial Institute-1640 (RPMI-1640) medium (Gibco, USA) supplemented with 15% fetal bovine serum (FBS) (Gibco, USA), 1% L-glutamine, and 1% penicillin/streptomycin at 37 °C in a 5% CO_2_ concentration.

Human BM-MSCs, collected from a 26-year-old healthy male donor, were obtained from the Institute of Royan Stem Cell Biology and Technology (Tehran, Iran) in passage 1 and cultivated in minimum essential medium eagle-alpha modification (α-MEM) (Bioidea, Tehran, Iran) containing 7% FBS, 1% L-glutamine, and 1% penicillin/streptomycin in a 37 °C humidified condition with 5% CO_2_ incubation. When the cells reached > 80% confluency, they were detached from the culture flasks and passaged using 0.25% trypsin-ethylenediaminetetraacetic acid (EDTA) (Gibco, USA). We observed the morphology of BM-MSCs at passage 2 using an inverted microscope to validate these cells. Furthermore, we analyzed the positive (CD90, CD73, and CD105) and negative (CD34, CD45, and CD14) expression of surface markers by flow cytometry (BD Biosciences, San Jose, CA, USA) [24].

### Exosome isolation

BM-MSCs at passages 3–6 were utilized for supernatant collection. These cells were seeded into a T75 culture flask, and upon reaching 90% confluency, the culture medium was replaced with a fresh FBS-free α-MEM medium. Subsequently, the cells were cultured for 72 h, and the culture medium was collected and used for exosome isolation. According to the manufacturer's protocols, MSC exosomes were isolated from the harvested medium using an Exocib isolation kit (Cibbiotech, Iran). Briefly, the medium harvested from BM-MSC was centrifuged at 3000 × *g* for 10 min to remove the cells and cellular debris. Next, the exosome precipitation reagent was added, and after vortexing for 5 min, the mixture was incubated overnight at 4 °C. The samples were then centrifuged at 3000 × *g* for 40 min, the supernatant was removed, and the final exosome pellet was resuspended in phosphate buffer saline (PBS). The isolated exosomes were stored at –20 °C for subsequent analysis.

### BM-MSC exosomes characterization

#### Dynamic Light Scattering (DLS)

DLS was used to assess the size distribution of the isolated exosomes. The samples were diluted in 100 μL of PBS and dispensed into a quartz cuvette. Subsequently, their sizes were measured at 630 nm using a Malvern Nano Zetasizer (UK).

#### Exosome surface markers analysis by flow cytometry

BM-MSC-derived exosomes were analyzed for specific exosome surface markers, CD63, CD9, and CD81, by flow cytometry (BD Bioscience, San Jose, CA, USA).

#### Transmission Electron Microscopy (TEM)

TEM was used to visualize the morphological structure of the isolated exosomes. The exosomes were fixed in 1% glutaraldehyde for 20 min, washed with PBS, and resuspended in PBS. A few drops were deposited onto a grid and stained with 1% uranyl acetate (PELCO; Ted Pella). Finally, their shapes were assessed using TEM (Zeiss-EM10C, Germany).

#### Measurement of the exosome concentration

The exosome concentration was determined using a bicinchoninic acid (BCA) protein assay kit (KIAZIST, Iran). Following the manufacturer's instructions, we prepared positive and negative controls, standard dilutions, and unknown sample dilutions and measured the absorbance at 570 nm. A standard curve was generated using a specific concentration of bovine serum albumin (BSA) protein, and the concentration of unknown samples was determined based on this curve.

### RNA extraction, c-DNA synthesis, and real-time PCR

JAK/STAT pathway gene expression levels were investigated in HL-60 cells treated with 0 and 100 µg/mL of exosomes for 24 h. Total RNA was extracted using 800 µL of TRizol™ Reagent (Qiagen, USA) according to the manufacturer’s instructions, and complementary DNA (c-DNA) was synthesized using a Thermo Scientific c-DNA kit (USA). To confirm the quantity and purity of the extracted RNA, a NanoDrop (Thermo Scientific, USA) was used to measure the OD at 260 nm and the 260/280 ratio, respectively. To estimate the expression of the mentioned genes, reverse transcription quantitative polymerase chain reaction (RT–qPCR) was performed in triplicate using SYBR™ Green Real-time PCR Master Mixes (Amplicon, Denmark) and primers specific to the genes of interest (Table 1). Glyceraldehyde-3-phosphate dehydrogenase (GAPDH) was used as a housekeeping gene to normalize the expression levels of the target genes. The Livak technique 2^^−∆∆Ct^ was used to calculate gene expression fold changes. The test was performed under the following conditions: denaturation at 95 °C for 5 min, followed by 40 cycles of 95 °C for 15 s, 60 °C for 15 s, and 72 °C for 15 s, and a final extension at 72 °C for 10 min [24].

**Table 1:** This table provides the primer sequences used in this study

Genes	primer	Product size	TM	Sequence (5’– > 3’)
JAK2	Forward Reverse	152	59.4 58.4	GGCAATGACAAACAAGGACAG AAGGAGGGGCGTTGATTTAC
STAT3	Forward Reverse	199	60.3 60.3	AGGAGGCATTCGGAAAGTATTG GGTTCAGCACCTTCACCATTAT
STAT5	Forward Reverse	177	59.4 61.3	CTGGCTAAAGCTGTTGATGGA TACATGGTCAGGGTTCTGTGG
GAPDH	Forward Reverse	70	59.5 60.5	ATGGGGAAGGTGAAGGTCG TAAAAGCAGCCCTGGTGACC

### Statistical analysis

All data are expressed as mean ± standard deviation (SD) of three triplicate measurements. Quantitative analysis of the flow cytometry results was performed using the FlowJo software (BD, USA). Statistical analyses were conducted using GraphPad Prism software (GraphPad Prism version 9.00). A t-test was employed to evaluate significant differences between the two groups. The level of significance was set at p < 0.05.

## Results

### Morphology and immuno-phenotyping of BM-MSCs

**Fig. 1:**
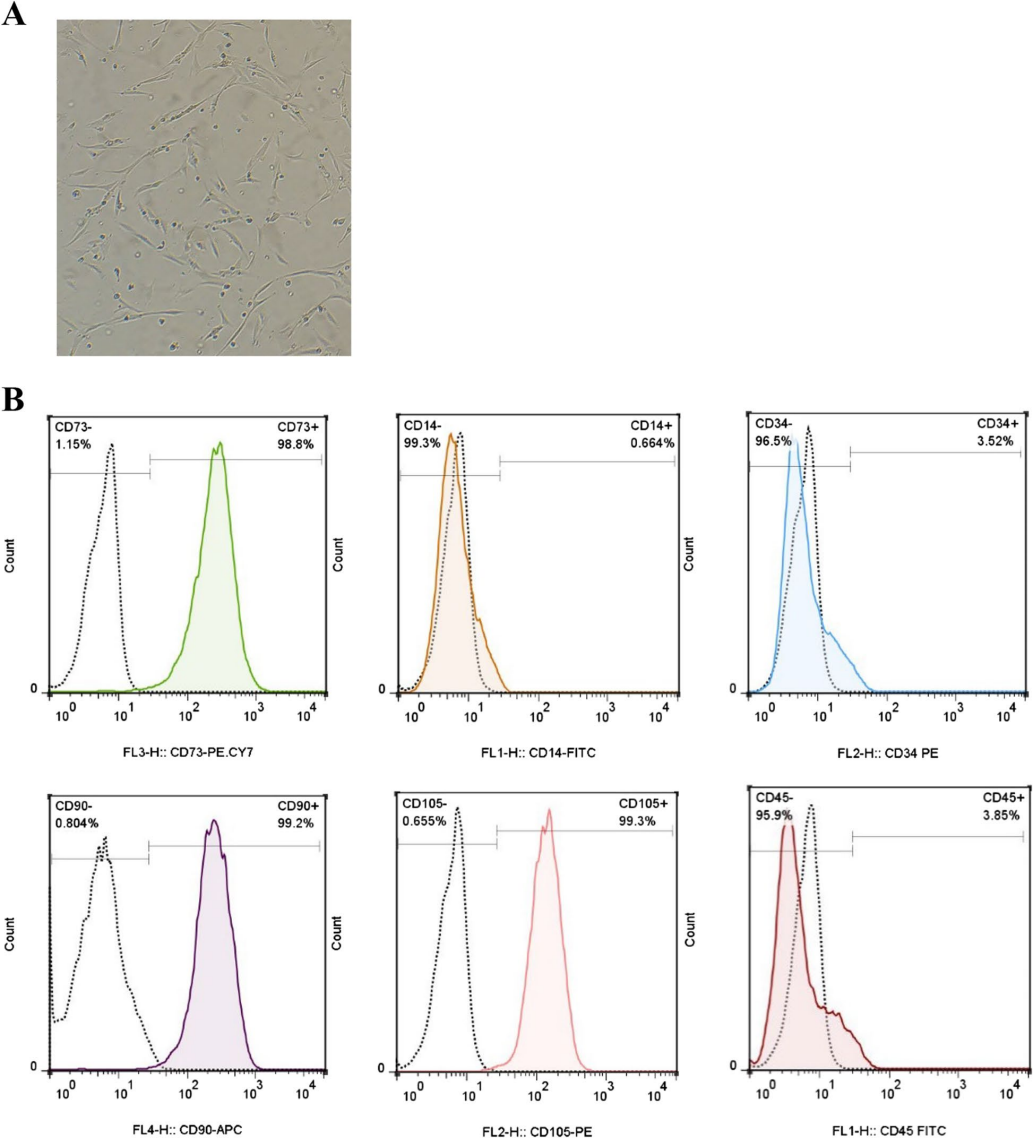
The identification and confirmation of BM-MSCs. **A** In passage 2, BM-MSCs demonstrated fibroblast-like morphology under an inverted microscope. **B** The flow cytometry was used to check the immune phenotype of BM-MSCs. BM-MSCs lacked CD45, CD34, and CD14, but expressed CD105, CD90, and CD73

Morphologically, the BM-MSCs appeared as a homogeneous population of spindle-shaped fibroblast-like cells that adhered to the cell culture flask (Fig. 1A). Furthermore, immunophenotyping of BM-MSCs using flow cytometry showed negative CD14, CD45, and CD34 expression and positive CD90, CD105, and CD73 (Fig. 1B).

### Characterization of isolated BM-MSC exosomes

**Fig. 2:**
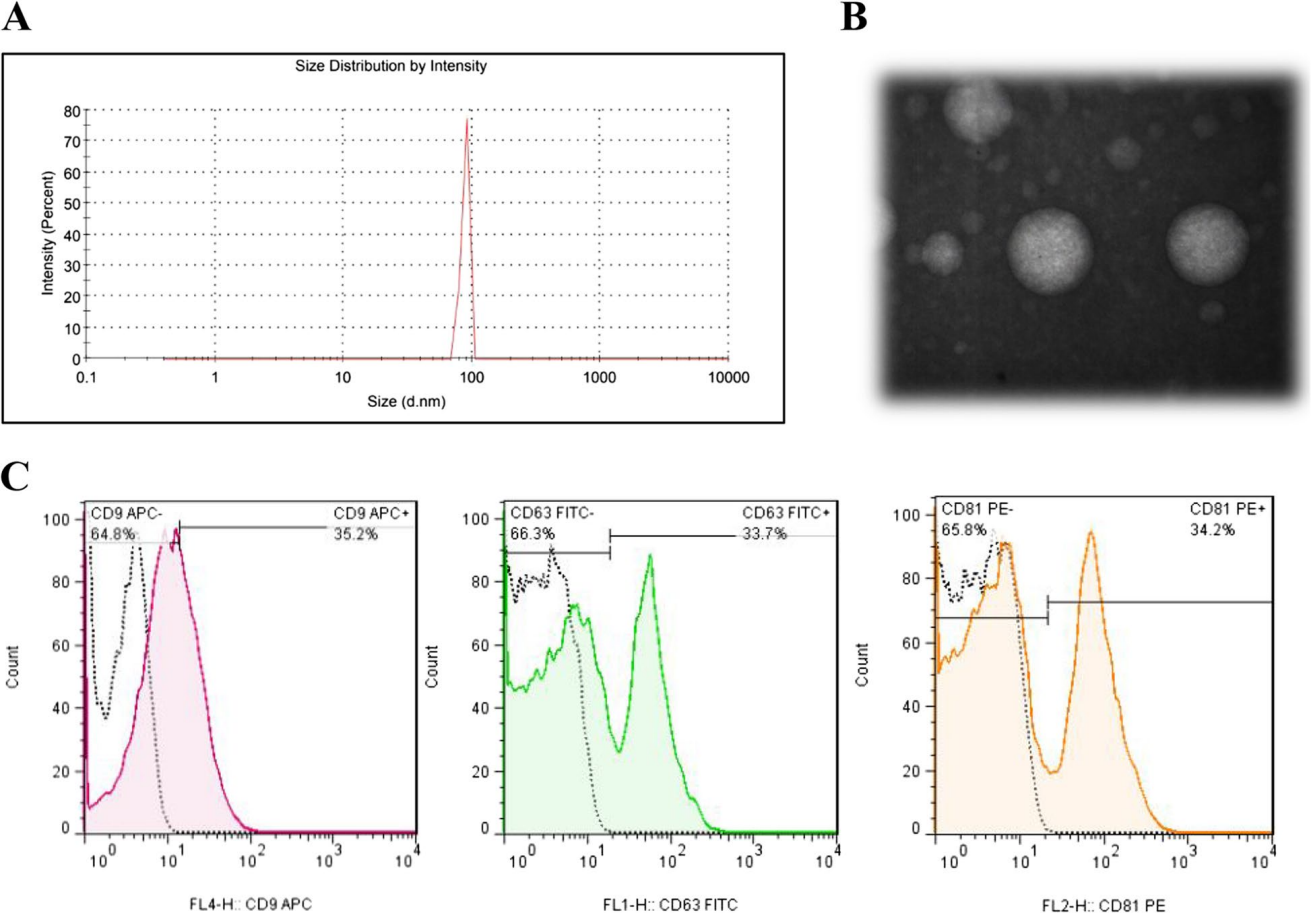
The identification and confirmation of BM-MSC exosomes. **A** The size of the isolated exosomes was determined using the DLS technique. The exosome sizes varied from 80–100 nm. **B** Morphology of isolated exosomes under TEM. The BM-MSC exosomes demonstrated a spherical-shaped morphology. **C** The expression of the CD63, CD81, and CD9 exosome-specific surface markers in BM-MSC exosomes was examined using flow cytometry

Exosomes isolated from BM-MSCs were confirmed by DLS, TEM, and flow cytometry. The DLS analysis (Fig. 2A) indicated that most exosomes had an 80–100 nm size range. TEM images (Fig. 2B) confirmed that most of the isolated vesicles exhibited a round morphology. Additionally, the isolated nanoparticles expressed specific surface markers CD81, CD63, and CD9 (Fig. 2C).

### Real-time PCR analysis

**Fig. 3:**
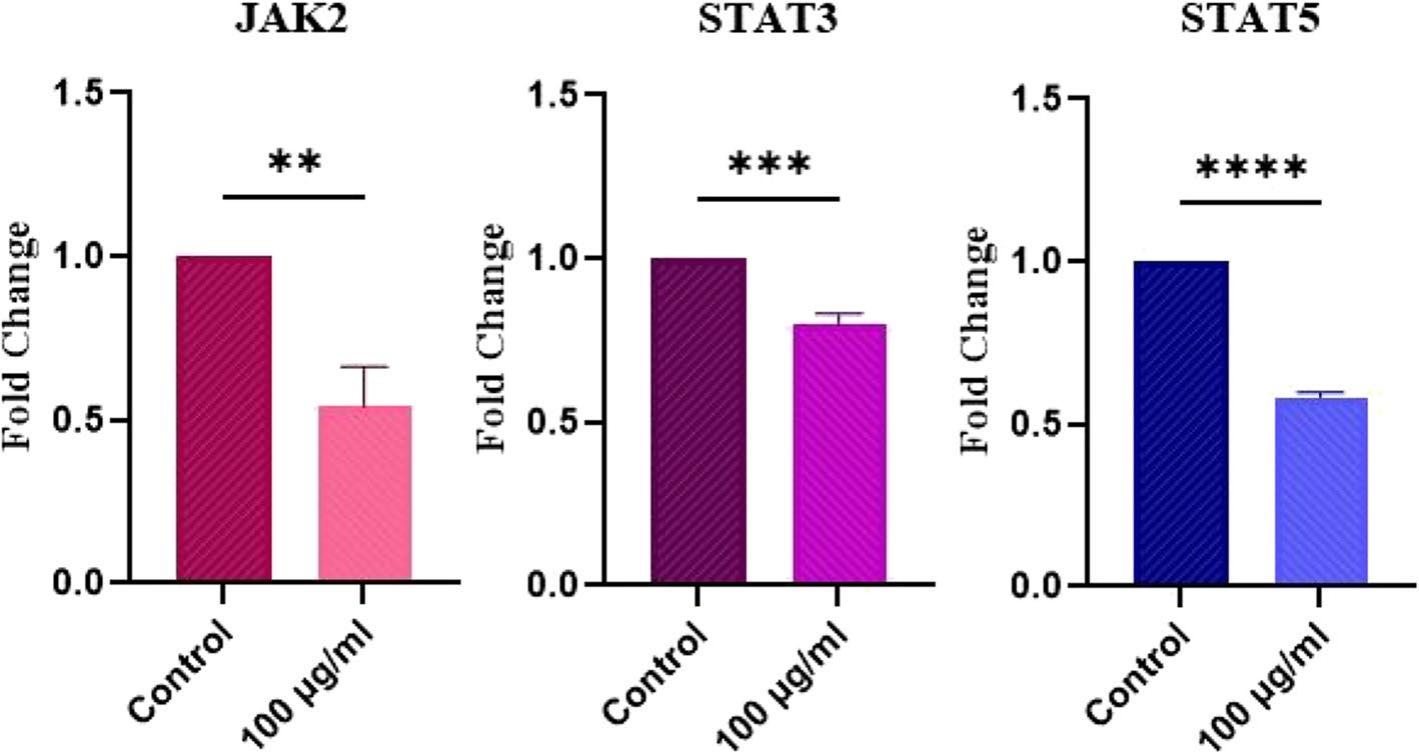
The impact of BM-MSC exosomes on the JAK/STAT gene expression of HL-60 cells. JAK2, STAT3, and STAT5 levels were notably reduced in HL-60 cells following treatment with 100 μg/mL of exosomes. The expression levels were normalized using the GAPDH housekeeping gene. Statistical significance was observed (*p < 0.05, **p < 0.01, ***p < 0.001, and ****p < 0.0001) compared to the control group (*n* = 3)

In our previous study, we used the MTT assay to find the proper dose and time of treatment with BM-MSC exosomes, the results of which indicated that the extracted exosomes had the most inhibitory effect on cell viability at a dose of 100 μg/mL in the first 24 h of treatment [24]. We examined the expression of JAK2, STAT3, and STAT5 in HL-60 cells by qRT-PCR after 24 h of treatment with 100 μg/mL of BM-MSC exosomes. The results shown in Fig. 3 indicate that the expression levels of JAK2, STAT3, and STAT5 genes were significantly decreased in treated cells compared with control cells.

## Discussion

Although intensive chemotherapy has long been the first-line treatment for AML, it is associated with significant morbidity and mortality in most patients [25]. These unfavorable outcomes highlight the need for alternative strategies with greater efficacy and fewer adverse effects to treat AML [7].

Stem cell-based therapies have emerged as potentially effective approaches to treat several diseases [26]. The unique qualities of MSCs, such as their potential for self-renewal and differentiation into various cell types, have made them the gold standard in adult stem cell medicine [26]. Research has explored the efficacy of MSCs in hematological malignancies and has suggested that these cells may have suppressive effects on leukemia and lymphoma [27, 28]. Among MSCs, BM-MSCs are considered the most suitable for clinical trials because of their accessibility and lack of ethical concerns [29]. It is now understood that MSCs can influence the behavior of tumor cells through paracrine signaling mechanisms rather than direct cell-to-cell contact [30]. There is increasing evidence that soluble factors secreted by MSCs, particularly exosomes, play a role in paracrine effects [31]. MSC-derived exosomes have demonstrated immunomodulatory, regenerative, and anti-inflammatory properties for treating human diseases [26]. Additionally, these exosomes are easily stored and transported and have low immunogenicity [32]. Therefore, MSC exosomes can potentially be valuable therapeutic tools for various diseases [33].

Interestingly, several studies have indicated an inhibitory role of MSC exosomes in AML. For example, Zhang et al. exposed THP-1 cells to BM-MSC-derived exosomes, which resulted in a decrease in viability and an increase in the apoptosis ratio [34]. Similarly, BM-MSC exosomes suppress proliferation, induce cell cycle arrest, and increase apoptosis of KG-1a cells [35]. Jiang et al. demonstrated that miR-7-5p from BMSC exosomes reduces survival and inhibits AML development by targeting OSBPL11 [36]. Additionally, Cheng et al. showed that MSC exosomes could inhibit AML cell growth through miR-23b-5p delivery [37]. Recent findings indicated that BM-MSC-derived exosomes increase the Th1/Th2 ratio and induce apoptotic cell death in AML cells [29]. In 2023, Wen et al. engineered MSC-derived exosomes with enhanced bone marrow homing and leukemic stem cell (LSC) targeting. These engineered vesicles, loaded with miR-34c-5p, selectively eliminated LSCs and impeded AML progression [38]. These results suggest that MSC exosome-based therapies are promising for treating AML. In this study, we evaluated the effect of BM-MSC exosomes on the expression of JAK/STAT signaling genes, which play a role in the proliferation and survival of AML cells.

Since our previous study demonstrated that the treatment with 100 μg/mL of BM-MSC exosomes led to a significant reduction in the survival and proliferation of AML cells and increased their ROS level and apoptosis [24], we chose this concentration for further gene expression analysis.

JAK2 is a kinase with crucial roles in the growth and development of hematological malignancies, particularly myeloproliferative neoplasms [39]. JAK2 hyperactivating mutations occur in AML [40], and a study by Ikezoe et al. demonstrated that p-JAK2 elevation in AML bone marrow samples was correlated with unsatisfactory clinical outcomes [41]. Notably, inhibition of JAK2 decreases AML LSCs’ growth while sparing normal stem cells in vitro and in vivo [42], suggesting that JAK2 may be a potential therapeutic target for AML.

Abnormal activation of JAK2/STATs leads to unrestricted proliferation of AML cells [43]. Interestingly, our findings revealed that treating AML cells with MSC-exosomes reduced the expression of JAK2 in AML cells, suggesting that BM-MSCs may obstruct JAK2 expression and activation in AML through exosome secretion.

STAT3 is a transcription factor that promotes carcinogenesis in most human malignancies [44]. Constitutive activation of STAT3 has been observed in many patients with AML, with those exhibiting such activity having lower disease-free survival rates than those without [45]. In addition, it has been demonstrated that the over-activation of JAK2/STAT3 is implicated in AML tumorigenesis, and targeting this pathway enhances the anti-tumor effects of arsenic trioxide in AML cells [23]. Furthermore, a study by Zhao et al. indicated that suppression of the JAK2/STAT3 pathway results in the inhibition of AML cell viability [46]. STAT3 is also involved in anti-apoptotic BCL2 gene expression [1]. Our previous study showed that BM-MSC exosomes decreased BCL2 expression, leading to apoptotic cell death in AML cells [24]. Here, we found that STAT3 was downregulated in response to treatment with MSC-exosomes, suggesting that these particles targeted STAT3 to suppress BCL2 expression and the subsequent activation of apoptosis in AML cells.

STAT5 is another member of the JAK/STAT signaling pathway that is constitutively activated in hematopoietic malignancies [47]. This molecule plays a crucial role in the various malignant characteristics of AML [48], and studies have shown that the inhibition of JAK2/STAT5 signaling can lead to apoptosis in AML cells [43].

Our findings suggest that MSCs utilize exosomes to inhibit the expression of this oncogene in AML cells, which may lead to anti-leukemic effects in AML.

Altogether, it is well established that the JAK/STAT signaling pathway contributes to leukemia initiation and progression, whose blockage may hamper leukemia growth and development. On the other hand, BM-MSC exosomes can suppress AML, and since our study demonstrated the inhibitory role of MSC-exosomes on the JAK/STAT pathway in AML cells, the use of JAK/STAT pathway inhibitors in combination with BM-MSC exosomes may be a novel approach for AML treatment.

In this study, we acknowledged the limitations of evaluating the impact of BM-MSC exosomes on the expression of JAK/STAT genes in AML patient samples and other cell lines. We recommend further research to explore the influence of BM-MSC exosomes on various AML cell lines and patient samples, as well as in mouse models (in vivo). Furthermore, it would be beneficial to assess the effects of BM-MSC exosomes on AML therapeutic responses by exposing exosome-cocultured leukemic cells to JAK/STAT signaling inhibitors and chemotherapy drugs.

## Conclusion

In this study, we examined the potential influence of MSC exosomes on the expression of JAK/STAT signaling genes in AML cells. Our results demonstrate a substantial reduction in JAK2, STAT3, and STAT5 expression in AML cells following treatment with BM-MSC exosomes. Considering the significance of the JAK/STAT pathway in AML cell proliferation and survival, the observed downregulation of JAK/STAT genes by BM-MSC exosomes could potentially impede AML cell proliferation and serve as a crucial aspect of targeted AML therapy.

## Data Availability

The datasets used and/or analyzed during the current study are available from the corresponding author upon reasonable request.

## Abbreviation

AML: Acute myeloid leukemia
MSC: Mesenchymal stem cell
BM: Bone marrow
DLS: Dynamic light scattering
TEM: Transmission electron microscopy
BCA: Bicinchoninic acid
JAK: Janus kinase
STAT: Signal transducer and activator of transcription
